# Impacts of Climate Change Interventions on Biodiversity, Water, the Food System and Human Health and Well‐Being

**DOI:** 10.1111/gcb.70444

**Published:** 2025-09-01

**Authors:** Pete Smith, Pramod K. Singh, Vedant P. Ballal, Francesco Cherubini, Julio Díaz‐José, Helena Duchková, Himangana Gupta, Masakazu Hori, Akihiko Ito, Shabana Khan, Marcos Llope, Maria Cristina Tirado, Luara Tourinho, Mariana M. Vale, Xiyan Xu, Harpalsinh Chudasama, Siri H. Eriksen, Daniel Mason‐D'Croz, Sui Chian Phang, Yash Srivastava, Tiff L. van Huysen, Taylor Ricketts, Mario Herrero, Paula A. Harrison, Pamela D. McElwee

**Affiliations:** ^1^ Institute of Biological & Environmental Sciences University of Aberdeen Aberdeen UK; ^2^ Institute of Rural Management Anand (IRMA) Anand India; ^3^ Industrial Ecology Programme, Department of Energy and Process Engineering Norwegian University of Science and Technology (NTNU) Trondheim Norway; ^4^ Faculty of Agriculture and Biological Sciences Universidad Veracruzana Xalapa Mexico; ^5^ Global Change Research Institute of the Czech Academy of Sciences Brno Czech Republic; ^6^ Faculty of Humanities Charles University in Prague Prague Czech Republic; ^7^ United Nations University – Institute for the Advanced Study of Sustainability Tokyo Japan; ^8^ Fisheries Resources Institute Japan Fisheries Research and Education Agency Yokohama Japan; ^9^ Graduate School of Agricultural and Life Sciences The University of Tokyo Tokyo Japan; ^10^ Indian Research Academy Delhi India; ^11^ Centro Oceanográfico de Gijón/Xixón, Instituto Español de Oceanografía (IEO‐CSIC) Gijón Spain; ^12^ School of Public Health, Institute of the Environment and Sustainability University of California, Los Angeles Los Angeles California USA; ^13^ Institute of Advanced Studies (IEA‐USP) University of São Paulo São Paulo Brazil; ^14^ Department of Ecology Federal University of Rio de Janeiro (UFRJ) Rio de Janeiro Brazil; ^15^ National Key Laboratory of Earth System Numerical Modeling and Application, Institute of Atmospheric Physics Chinese Academy of Sciences Beijing China; ^16^ Aga Khan Rural Support Programme Delhi India; ^17^ Faculty of Landscape and Society Norwegian University of Life Sciences Ås Norway; ^18^ Department of Global Development, College of Agriculture and Life Sciences Cornell University Ithaca New York USA; ^19^ Agricultural Economics and Rural Policy Group Wageningen University and Research Wageningen the Netherlands; ^20^ The Nature Conservancy London UK; ^21^ Sambodhi Research and Communications Noida India; ^22^ Intergovernmental Science‐Policy Platform on Biodiversity and Ecosystem Services (IPBES) Bonn Germany; ^23^ Gund Institute for Environment University of Vermont Burlington Vermont USA; ^24^ Rubenstein School for Environment and Natural Resources University of Vermont Burlington Vermont USA; ^25^ UK Centre for Ecology & Hydrology Lancaster UK; ^26^ School of Environmental and Biological Sciences Rutgers University New Brunswick New Jersey USA

**Keywords:** biodiversity, climate change, food, human health and well‐being, IPBES, Nexus, synergy, trade‐off, water

## Abstract

Climate change threatens biodiversity, water, food and human health and well‐being. Rapid, sustained mitigation and adaptation actions can benefit all these elements of the nexus. Key transitions in energy, land and marine ecosystems, urban areas, industry and society are essential for climate change mitigation, adaptation and sustainable development. These transitions require interdisciplinary research, policy support and societal engagement. Here we present an assessment of 69 response options, a subset of which (15) was used in the climate change chapter of the IPBES Nexus Assessment. We show that the majority of climate change response options for land, oceans and ecosystems, settlement and infrastructure, industrial and societal system transitions have broadly positive impacts across the nexus. However, energy system transitions show more apparent trade‐offs. Most of these impacts result from energy infrastructure that would also be required for fossil fuel‐based systems and should be compared to the far more damaging consequences of continued fossil fuel use. Transitioning to cleaner, renewable energy sources reduces these risks and offers significant improvements across the nexus by reducing climate change impacts. Of the 69 response options assessed, 59% have entirely positive effects, or at least no negative effects, across all nexus elements and can be considered as low‐risk, immediately actionable options. The remaining 41% show either negative or variable impacts on at least one nexus element. However, this does not render them unviable; rather, their implementation must be carefully managed. Where impacts are variable, strategies should be tailored to ensure positive outcomes; where trade‐offs are unavoidable, efforts should focus on minimising negative effects and maximising synergies. Our findings suggest that prioritising policies that address the interconnected challenges of climate change, biodiversity loss, land degradation, pollution, food insecurity, access to clean water, energy for all and sustainable development will deliver more effective and equitable climate action.

## Graphical abstract

Climate change threatens biodiversity, water, food, and human health and well‐being. Rapid, sustained mitigation and adaptation actions can benefit all these elements of the nexus. Of the 69 response options assessed in this study, 59% have entirely positive effects, or at least no negative effects, across all nexus elements and can be considered as low‐risk, immediately actionable options. The remaining 41% show either negative or variable impacts on at least one nexus element. Where impacts are variable, strategies should be tailored to ensure positive outcomes; where trade‐offs are unavoidable, efforts should focus on minimising negative effects and maximising synergies.

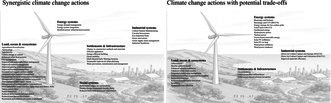

## 1 Introduction

The world is facing multiple challenges across the nexus of climate, biodiversity, water, food systems and human health and well‐being. We are facing an ever‐worsening climate emergency, with the global mean temperature in 2024 surpassing the 1.5°C (above pre‐industrial levels) goal of the Paris Agreement, with uncertain and unprecedented extreme events threatening lives, infrastructure and ecosystems the world over (Copernicus 2025). At the same time, we face a global water crisis; over 700 million people, or 1 in 10 people, lack access to clean water (World Vision 2024). We are facing a global food system crisis; 783 million people on the planet are facing chronic hunger, with 7 million deaths per year from diets low in whole grains, fruits, nuts and seeds (Afshin et al. 2017) and with many more consuming unhealthy diets (World Food Programme 2024). We are in the midst of a nature emergency; 1 million species are threatened with extinction, and the global rate of decline in species has accelerated to an unprecedented rate in human history (IPBES 2019a). We are facing a range of global health and well‐being challenges, including antimicrobial resistance, increases in impacts of climate change on a range of health issues, a rise in non‐communicable diseases such as cardiovascular conditions, diabetes and cancer and inadequate prevention and preparedness for infectious diseases and pandemics (WHO 2024; Alders et al. 2024). Due to ambient air pollution, primarily associated with fossil fuel use and biomass burning, nearly 7 million people die each year, and many more are subject to health damage, and the number of deaths from air pollution has risen 66% in the past two decades (Vohra et al. 2021; Fuller et al. 2022). Mental illnesses are also a serious concern, with an increase in mental health disorders, including depression and anxiety, especially in low‐ and middle‐income countries with insufficient mental health services and health inequities (WHO 2024).

The adverse impacts of climate change on biodiversity, ecosystem services, freshwater resources, food security and human health are well documented (IPCC 2022; Pörtner et al. 2021) and are often affected by common drivers. In addition to its direct impacts, climate change is also acknowledged as a ‘threat multiplier’ because it amplifies existing vulnerabilities in critical sectors, intensifying challenges such as biodiversity loss, water scarcity, acute food insecurity and health crises (IPCC 2022). These cascading impacts emerge from extensive human activities that significantly alter terrestrial, freshwater, coastal and marine ecosystems and further influence climate variability (IPCC 2019c).

Climate change both impacts and is impacted by all other nexus elements: biodiversity, water, food and human health and well‐being. For example, the healthcare sector is responsible for 5% of global greenhouse gas (GHG) emissions (Watts et al. 2021). Healthy ecosystems supporting abundant biodiversity have been shown to be more resilient to climate change (Shin et al. 2022) and to provide effective nature‐based solutions for climate change mitigation and adaptation (Smith et al. 2022; Huang, Shen, et al. 2022; Xu et al. 2022). Freshwater resources are threatened by drought and excessive precipitation, with such extreme events becoming more prevalent under climate change (IPCC 2022), while perturbations to the water cycle have impacts on climate (IPCC 2021). Similarly, food security is threatened by climate change, including extreme weather events (IPCC 2022), whereas the global food system is responsible for one third of anthropogenic GHG emissions (Crippa et al. 2021) and hence has large opportunities for climate change mitigation (Babiker et al. 2022).

Response options to tackle climate change fall into two categories: mitigation and adaptation. The IPCC definition of mitigation is ‘A human intervention to reduce the sources or enhance the sinks of GHGs’ (IPCC 2021). According to the IPCC definition, adaptation is ‘the process of adjustment to actual or expected climate and its effects. In human systems, adaptation seeks to moderate or avoid harm or exploit beneficial opportunities. In some natural systems, human intervention may facilitate adjustment to expected climate and its effects’ (IPCC 2021). ‘Climate action’ comprises climate change mitigation and adaptation (IPCC 2018). Response options can deliver mitigation, adaptation or both, with increasing attention being paid to the urgent need for integrating mitigation and adaptation in actions founded in ecosystem stewardship and social justice, in order to advance sustainable, climate‐resilient development for all (Schipper et al. 2022).

Sector‐specific policies and response options often yield suboptimal outcomes, exacerbating existing challenges like resource depletion and biodiversity loss (Aggestam et al. 2023; Newell et al. 2019; Tudose et al. 2021). Alternatively, approaches that consider multiple challenges together augment the co‐benefits of action to address climate change. For example, a transition to low‐GHG energy sources mitigates climate change and improves air quality and public health (IPCC 2022). However, potential (and actual) trade‐offs need to be avoided or minimised, especially those that could threaten human well‐being or food security and escalate resource competition, or result in ecological degradation (Pörtner et al. 2021; Smith et al. 2020).

There has been a recognition that many of the global challenges described above are linked, and that potential solutions for one challenge could either contribute positively to solving others or could create trade‐offs with other challenges. In 2019, the IPCC published a Special Report on Climate Change and Land (IPCC 2019a). That report focussed on response options to address climate change on land, specifically the impacts of interventions for climate change mitigation and adaptation, on halting or reversing land degradation and desertification and on food security. Smith et al. (2020) summarised the cross‐sectoral findings of that report. In 2021, the 8th session of the IPBES Plenary approved the undertaking of a thematic assessment report on the interlinkages among biodiversity, water, food and health in the context of climate change, known as the ‘Nexus Assessment’. The outputs in 2021 from an IPBES and IPCC co‐sponsored workshop on biodiversity and climate change (Pörtner et al. 2021), with work contributing to that workshop report published by Shin et al. (2022) and Smith et al. (2022), represented a contribution to the Nexus Assessment. The Nexus Assessment was approved by the IPBES Plenary in December 2024 (IPBES 2024) and examines the ‘nexus’ impacts of interventions on biodiversity, water, food, human health and well‐being and climate.

The authors of this paper were all experts working on the IPBES Nexus Assessment and contributed to Chapter 5.5 entitled ‘Options for delivering sustainable biodiversity‐related approaches to climate change, adaptation and mitigation, including relevant aspects of the energy system’. In this paper, we report on work undertaken to compile and assess a long‐list of 69 response options predominantly implemented to address climate change, only a subset of which (15) were reported in the final chapter, and assess and discuss the co‐benefits and trade‐offs these response options have on biodiversity, water, the food system and human health and well‐being.

## 2 Materials and Methods

Chapter 18 of the IPCC WGII's Sixth Assessment Report (IPCC 2022) identifies five domains for rapid transitions to facilitate climate change mitigation, adaptation and sustainable development: (i) land, oceans and ecosystems; (ii) energy; (iii) settlement and infrastructure; (iv) industrial and (v) societal systems (Schipper et al. 2022). These system transitions are used for organising the response options in this study.

### 2.1 Land, Oceans and Ecosystems Transitions

Land, oceans and ecosystems are experiencing significant transitions driven by climate change, with implications for resilience and nature's contributions to people (NCP) (IPBES 2019b). At the same time, many response options exist within agriculture, forestry and other land uses, both for adaptation and mitigation, that can drive transitions towards sustainability (Nabuurs et al. 2022).

### 2.2 Energy System Transitions

Energy systems require shifts towards low carbon (including renewable) energy sources, improved energy efficiency and carbon capture and storage to meet climate change mitigation objectives and abate emissions of CO_2_ from fossil fuels (IEA 2019). Ongoing energy transitions respond to climatic and non‐climatic considerations, with sustainable development priorities driving change (Clarke et al. 2022).

### 2.3 Settlement and Infrastructure System Transitions

Urban and rural settlements and infrastructure play a crucial role in climate‐resilient futures but face risks from climate stresses (Davidson et al. 2019). Enhancing urban resilience can be achieved through investments in disaster risk reduction, climate‐resilient green infrastructure and updated building codes (Dodman et al. 2022). Integrating nature‐based solutions and green infrastructure can enhance resilience and support economic development (Shaneyfelt et al. 2017; Prado et al. 2024).

### 2.4 Industrial System Transitions

Industrial system transitions comprise dematerialisation and decarbonisation (Petrides et al. 2018; Worrell et al. 2016), supported by effective governance, enlightened policies, green supply chains, strong regulations and empowering corporate strategies (Singh and Chudasama 2021).

### 2.5 Social System Transitions

Societal system transitions focus on changing behaviours, attitudes, values and consciousness across society (De Witt et al. 2016; Schipper et al. 2022), as well as social, institutional and technological change. Such transitions involve enabling conditions for just individual and collective actions, including inclusive governance, civic engagement and shifting development paradigms and socio‐political power imbalances (Schipper et al. 2022).

In preparation for the climate change subchapter (Singh et al. 2024) of the IPBES Nexus Report (IPBES 2024), the author team drew up a non‐exhaustive long list of 69 options, representative of the five system transitions described above, drawn mainly from previous IPCC and IPBES reports (Babiker et al. 2022; IPCC 2018; McElwee et al. 2020; Pörtner et al. 2021; Schipper et al. 2022; Smith et al. 2020; Smith et al. 2022), and supplemented by others identified by the author team. It is that collection of 69 response options that is assessed here, only 15 of which were fully assessed and reported upon in Singh et al. (2024).

The response options considered under each of the system transitions (some of which overlap to a certain extent) and their descriptions, adopted by the author team, are given below in Tables 1, 2, 3, 4, 5. Note that these are not official definitions since various bodies (e.g., IPCC, IPBES, UNEP, FAO and many others) use different definitions; these are the working descriptions used by the author teams when assessing the literature.

**TABLE 1:** Land, Ocean and Ecosystems Transition response options.

Response option	Description
Afforestation	The process of establishing a forest or stand of trees in an area where there was in a non‐forest biome where there was no previous tree cover
Agricultural diversification	Diversifying production to crops or livestock that are not mainstream, for example, growing heritage varieties of crops or fruit or different breeds of farm animal
Agroecology	Designing and managing agricultural and food systems using ecological and social contexts and principles to support sustainable agricultural production, minimise negative environmental impacts of production and secure nature's contributions to people
Agroforestry	Land management system that integrates trees and shrubs into agricultural landscapes to create environmental, economic and social benefits, including alley cropping, silvopasture, riparian zones and windbreaks
Biochar addition to soil	The addition of carbon‐rich material produced by heating organic matter, such as plant or animal waste, in an oxygen‐limited environment through a process called pyrolysis to the soil
Carbon storage in seabed	Methods to place organic materials on the seabed. Organic carbon can be preserved in marine sediments through natural processes like burial, sorption (carbon uptake by mineral surfaces), and molecular transformation. These processes help protect organic matter from degradation
Enhanced mineral weathering	A process that accelerates the natural weathering of minerals to capture and store carbon dioxide (CO_2_) from the atmosphere. This method involves spreading finely ground silicate rocks, such as basalt, onto land or ocean surfaces. The chemical reactions between these minerals, water and CO_2_ result in the formation of stable carbonates, which can store carbon for long periods
Enhanced urban food systems	Enhancing the processes and infrastructure involved in feeding urban populations, for example, growing food within cities through community gardens, rooftop farms and vertical farming
Fire management	Prevention of wildfires or using fire as a tool to maintain and restore the health of ecosystems
Improved and sustainable forest management	Practices that enhance the health and productivity of forests while ensuring their long‐term sustainability, for example, by selective logging, optimised harvest cycles, adaptive management
Improved cropland management	Improving the management of land used for arable crop production, for example, through reduced intensity tillage, residue management, improved rotations, improved nutrient delivery to crops
Improved grazing land management	Improving the management of land used for the grazing of livestock, for example, through appropriate stocking density, improved and diverse sward and improved nutrient delivery to grass
Improved livestock management	Improved management of livestock, such as breed improvements, dietary additives and better disease control
Increased soil organic carbon content	The process of enhancing the amount of carbon stored in the soil in the form of organic matter, such as decomposed plant and animal materials. This can be achieved through various sustainable agricultural practices
Integrated coastal zone management	Integrated coastal zone management (ICZM) is a comprehensive and sustainable approach to managing coastal areas, balancing environmental, social and economic concerns. The mitigation potential of ICZM can be difficult to quantify as it involves a wide range of activities across different coastal ecosystems and regions. The mitigation potential of ICZM lies primarily in the conservation and restoration of blue carbon coastal ecosystems: mangroves, seagrasses and salt marshes. If properly managed, ICZM can also reduce greenhouse gas emissions from coastal infrastructure, prevent carbon release from coastal degradation and support the deployment of renewable energy sources. Note overlap with management of coastal and marine ecosystems
Integrated water resource management	Holistic approach that promotes the coordinated development and management of water, land and related resources. The goal is to maximise economic and social welfare without compromising the sustainability of vital ecosystems
Management for biodiversity and ecosystem services	Management for biodiversity and ecosystem services involves strategies and practices aimed at conserving and enhancing the variety of life forms (biodiversity) and the benefits that ecosystems provide to humans (ecosystem services). It often involves Ecosystem‐Based Management (EBM), adaptive management, conservation and restoration, sustainable use of resources, strong policy and governance and community involvement, for climate change mitigation and adaptation
Management of coastal and marine ecosystems	Managing coastal and marine ecosystems involves a variety of strategies aimed at preserving and restoring these vital environments. Note overlap with Integrated Coastal Zone Management, and sustainable ocean fisheries
Management of food supply chains	Improved management of food supply chains to reduce GHG emissions and reduce waste. Interventions can be at all stages of the supply chain from production through to end use and waste management
Management of invasive species/encroachment	Managing invasive species and encroachment involves several strategies to prevent, control and eradicate these species to protect ecosystems, agriculture and human health through, for example, prevention, early detection and rapid response, mechanical, biological and chemical control, integrated pest management
More sustainable ocean fisheries, aquaculture and dietary shifts	Harvesting ocean products more sustainably, establishing sustainable aquaculture and dietary shifts away from fish and other marine foods, the harvest of which damage the environment
Nature conservation	The practice of protecting and managing the natural environment to ensure the sustainability of ecosystems, species and natural resources. It involves a range of activities aimed at preserving biodiversity, restoring habitats and mitigating the impacts of human activities on the environment
Nature‐based ILK	Indigenous and local knowledge (ILK) plays a crucial role in nature‐based solutions (NbS) by integrating traditional practices and cultural insights into modern conservation and sustainability efforts. ILK, also known as Traditional Ecological Knowledge (TEK), encompasses knowledge and practices passed down through generations, informed by cultural memories, sensitivity to environmental changes and values like reciprocity and kinship with nature
Reduced conversion of grassland to cropland	Reduced conversion of grassland to cropland
Reduced deforestation and degradation	Reducing deforestation rates and reducing forest degradation to enhance carbon sequestration and biodiversity
Reduced post‐harvest food losses	The reduction in quantity and quality of agricultural produce from the time of harvest until it reaches the consumer. This loss can occur at various stages, including harvesting, handling, storage, processing and transportation
Reduced soil erosion	Reducing the erosion of soils through, for example, establishing a cover of grass, shrubs or trees the roots of which helps hold the soil together, mulching, contour farming, terracing, cover crops, erosion control mats
Reforestation and forest restoration	Reforestation involves the natural or intentional regeneration of tree cover in areas where forests have been lost and forest restoration encompasses a broader range of activities aimed at returning a forest to a healthy state, through, for example, maintaining tree diversity and forest structure or reducing invasive species
Restoration and reduced conversion of peatlands	Reducing the conversion of, and restoring degraded peatlands through measures to return degraded peatlands to their natural state, improving their ecological function and carbon storage capacity
Rewilding	An ecological restoration approach aimed at increasing biodiversity and restoring natural processes by reducing human influence on ecosystems. It involves reintroducing native species, especially keystone species, and allowing natural processes to shape the landscape
Sustainable intensification	An agricultural approach aimed at increasing food production from existing farmland while minimising environmental impact and promoting social and economic benefits

**TABLE 2:** Energy system transition response options.

Response option	Description
Bioenergy and biofuels	Bioenergy is a form of renewable energy derived from biomass which are combusted, digested (usually by anaerobic digestion) are thermochemically converted. Biofuel usually refers to liquid fuels used for transportation. This response option also considers the impacts of growing the biomass
Bioenergy and CCS (BECCS)	As for bioenergy, but with the carbon dioxide produced from combustion being stored is geological reservoirs, such as depleted oil or gas reservoirs, saline aquifers or seams or basalt
Energy demand management	Energy demand management, also known as demand‐side management (DSM), involves strategies to modify consumer demand for energy, included reducing overall demand and encouraging consumers to use less energy during peak hours or shift their usage to off‐peak times
Energy storage for low‐carbon grids	Energy storage, crucial for integrating renewable energy sources into low‐carbon grids, can take the form or chemical storage (batteries), gravity storage (pumped hydro storage—see also hydroelectric power, large weights), rotational kinetic energy (flywheels), thermal storage and hydrogen storage
Energy system integration	Energy system integration involves creating a more interconnected and coordinated energy network to optimise the use of renewable resources and enhance efficiency
Fossil fuels with CCS	The combustion of fossil fuels, for example, coal, oil, gas, but with the carbon dioxide produced from combustion being captured and durably stored in geological reservoirs, such as depleted oil or gas reservoirs, saline aquifers or seams or basalt
Geothermal energy	Geothermal energy is a renewable energy source that harnesses heat from within the Earth. It includes deep geothermal where the energy originates from the Earth's core, and geothermal heat pumps that use the stable temperatures near the Earth's surface to heat and cool buildings
Hydroelectric power	Hydroelectric power, or hydropower, is a renewable energy source that generates electricity by using the energy of flowing or falling water. It can include flow from dams and reservoirs, run‐of‐river systems and pumped storage
Nuclear power	Nuclear power is a method of generating electricity using the energy released from nuclear reactions, usually from nuclear fission of (usually) uranium‐235 or plutonium‐239. The response option also considers involved the mining of ores and also the disposal of radioactive waste
Ocean‐based renewable energy	This response option considers the wider range of renewable energy generation techniques, in addition to offshore PV and offshore wind, such as wave energy, tidal energy, ocean thermal energy conversion (OTEC) and salinity gradient energy (also known as blue energy)
Resilient power infrastructures/systems	Resilient power infrastructures are designed to withstand and quickly recover from disruptions, ensuring a reliable supply of electricity and can include multiple power sources and backup generators to maintain power during outages, microgrids (small‐scale power grids that can operate independently or in conjunction with the main grid), energy storage (see above), smart grids that utilising advanced technologies to monitor and manage the grid in real‐time
Solar PV (offshore)	Solar photovoltaic (PV) technology is a form of renewable energy that converts sunlight directly into electricity using semiconductor materials. This response option considers solar PV panels placed in arrays offshore
Solar PV on land	Solar photovoltaic (PV) technology is a form of renewable energy that converts sunlight directly into electricity using semiconductor materials. This response option considers solar PV panels placed in arrays on land (including those of build infrastructure)
Wind power (offshore)	Wind power is a form of renewable energy that converts the kinetic energy of wind into mechanical or electrical energy. This response option considers turbines sited offshore
Wind power (onshore)	Wind power is a form of renewable energy that converts the kinetic energy of wind into mechanical or electrical energy. This response option considers turbines sited on land

**TABLE 3:** Settlement and infrastructure system transition response options.

Response option	Description
Change in construction methods and materials	Low‐impact construction materials and methods focus on reducing the environmental footprint of building projects while promoting sustainability and efficiency, including, for example, using recycled materials and low‐emission concrete, installing green rooves and incorporating passive solar design
Efficient appliances	Efficient appliances are designed to use less energy and resources while performing their intended functions. These appliances help save money on energy bills, reduce greenhouse gas emissions and contribute to a more sustainable future
Electromobility	Electromobility, or e‐mobility, refers to the use of electric propulsion for various types of transportation, including cars, buses, trucks, bicycles, ships and ferries
Energy‐efficient building	Energy‐efficient buildings are designed to minimise energy consumption while maintaining a comfortable and healthy indoor environment. These buildings incorporate various technologies, materials and design strategies to achieve high levels of energy efficiency
Green mobility	Green mobility refers to the adoption of environmentally sustainable transportation methods and solutions to reduce the carbon footprint and environmental impact of moving people and goods, through, for example, electric vehicles, public transportation and encouraging active transport (walking, cycling, etc.)
Multi‐hazard early warning systems	Multi‐hazard early warning systems (MHEWS) are designed to provide timely and effective information about multiple types of hazards, enabling individuals, communities and governments to take action to reduce disaster risks before hazardous events occur, for example, flood/drought early warning systems
Sustainable land‐use and urban planning	Sustainable land use and urban planning aim to create cities and communities that are environmentally friendly, economically viable and socially inclusive, through, for example, the efficient use land to balance development and conservation, green infrastructure (incorporating natural elements like parks, green roofs and urban forests) and promoting higher density development to reduce urban sprawl, preserve natural landscapes and make public transportation more viable
Urban green/blue infrastructure	Urban green/blue infrastructure refers to the integration of natural and semi‐natural elements into urban environments to address environmental, social and economic challenges. This approach combines green spaces (like parks and gardens) with blue elements (such as rivers, ponds and wetlands) to create sustainable and resilient urban areas
Urban nature‐based solutions (ecopolis)	Urban nature‐based solutions (NbS) involve integrating natural elements into city environments to address various societal challenges. These solutions include actions like planting trees, creating parks, installing green roofs and restoring wetlands. They aim to enhance biodiversity, improve air quality, mitigate climate change impacts and make urban areas more resilient and sustainable
Waste prevention, minimisation and management	Waste prevention, minimisation and management are crucial strategies for reducing the environmental impact of waste and promoting sustainability. Waste prevention involves actions taken to avoid generating waste, waste minimisation focuses on reducing the amount of waste produced through various practices, and waste management involves the proper handling, treatment and disposal of waste to minimise its environmental impact

**TABLE 4:** Industrial system transition response options.

Response option	Description
Carbon‐neutral manufacturing	Carbon‐neutral manufacturing aims to eliminate or offset the carbon emissions produced during the manufacturing process, including, for example, energy efficiency, renewable energy, carbon offsetting, circular economy practices and supply chain management
Circular bioeconomy	A circular bioeconomy focuses on using renewable biological resources to create sustainable products and services while minimising waste and environmental impact. This approach integrates principles of the circular economy with the sustainable management of biological resources
Direct Air Carbon Capture and Storage (DACCS)	Direct Air Carbon Capture and Storage (DACCS) is a technology designed to capture carbon dioxide (CO_2_) directly from the atmosphere and store it in geological formations or use it in various products
Direct Air Carbon Capture and Utilisation (DACCU)	Direct Air Carbon Capture and Utilisation (DACCU) is a technology that captures carbon dioxide (CO_2_) directly from the atmosphere and uses it to create valuable products, rather than storing it underground as in Direct Air Carbon Capture and Storage (DACCS)
Eco‐industrial parks	Eco‐industrial parks (EIPs) are industrial areas where businesses collaborate with each other and the local community to reduce waste and pollution, efficiently share resources and achieve sustainable development. The concept is based on industrial ecology, where the waste or by‐products of one company can become the input for another, creating a closed‐loop system
Green innovations (innovations in processes, techniques, systems and products)	Green innovations refer to new technologies, practices and products that aim to reduce environmental impact and promote sustainability, including renewable energy, sustainable materials, water conservation, waste management and green transportation
Green supply chain management	Green Supply Chain Management (GSCM) integrates environmentally sustainable practices across the entire supply chain, from product design and material sourcing to manufacturing, transportation and end‐of‐life management
Industrial Symbiosis	Industrial symbiosis is a collaborative approach where different industries work together to use each other's by‐products and waste materials as resources. This concept is a subset of industrial ecology and aims to create a more sustainable and efficient industrial system
Materials efficiency	Materials efficiency involves using fewer materials to produce goods and services, thereby reducing waste and conserving resources. It includes features such as lightweight design, longer lasting and recyclable products and efficient manufacturing processes

**TABLE 5:** Social system transition response options.

Response option	Description
Behavioural nudges for sustainability	Behavioural nudges are subtle interventions designed to influence people's behaviour in a predictable way without restricting their freedom of choice. These nudges can be highly effective in promoting sustainable practices and include setting eco‐friendly choices as the default option, changing social norms by highlighting positive behaviours of others that can encourage individuals to follow suit, providing real‐time feedback on energy or water usage can help individuals understand their consumption patterns and motivate them to reduce usage, commitment devices that encourage people to make public commitments to sustainable behaviours and gamification of sustainable actions
Dietary change (sustainable healthy diets)	Dietary change to sustainable healthy diets involves a transition to diets that promote individuals' health and well‐being which have low environmental pressure and impact, are accessible, affordable, safe, equitable and culturally acceptable. They tend to be rich in fruits and vegetables and require a decrease in consumption of livestock products in over‐consuming populations
Reduced food waste (consumer and retailer)	Reducing food waste is essential for addressing environmental, economic and social challenges. For the consumer this involves smart shopping to avoid impulse buys to prevent over‐purchasing, proper storage, understanding labels and creative cooking. For the retailer this involves advanced inventory systems, partnering with local food banks and charities to donate surplus food, consumer education and waste reduction practices

Each option selected contributes positively to either climate change mitigation, climate change adaptation or both. We conducted an extensive literature review to quantify the contribution of each climate change response option to climate change mitigation, climate change adaptation, biodiversity, water, food and human health and well‐being.

For each response option, the impact on each nexus element (climate change mitigation, climate change adaptation, biodiversity, water, food and human health and well‐being) was scored on a 7‐point scale, in a similar way to the expert elicitation/literature review in Herrero et al. (2020) and Chrysafi et al. (2022). The scoring categories, thresholds and means of determining the overall scores are described in the Supporting Information.

## 3 Results

### 3.1 Land, Ocean and Ecosystems Transition Response Options

Twenty‐two of the 31 (71%) response options under the Land, Ocean and Ecosystems Transition category have no negative impacts on any of the elements for which they can be scored. Four (13%) response options cannot be scored for global mitigation potential; one (3%; reduced soil erosion) can have either a positive or negative global mitigation potential, and ten (32%) cannot be scored for global adaptation potential, but all other response options (68%) have positive impacts of climate change mitigation and adaptation. Sixteen of the 31 options (52%) also contribute positively to each of the nexus elements of biodiversity, water, food system and human health and well‐being. A further eight (26%) response options have insufficient data for at least one element, but otherwise only contribute positive impacts. Six (19%) of the response options have a potential negative impact on at least one of biodiversity, water, food system and human health and well‐being, with a further four (13%) having variable impacts (both positive and negative impacts reported in the literature depended on context) on at least one element (Table 6).

**TABLE 6:** Impacts of climate change response options under the Land, Ocean and Ecosystems Transition category on biodiversity, water, food system and human health and well‐being.

Response option	Mitigation impact (GtCO2e/yr)	Adaptation impact (millions of people)	Biodiversity impact	Water impact	Food system impact	Health and well‐being impact
Afforestation	1.5–10.1	nd	− or L+	Variable	H−	L+
Agricultural diversification	> 0	> 25	H+	nd	H+	M+
Agroecology	1.4–2.3	> 25	M+	H+	L+	H+
Agroforestry	0.1–5.7	2300	H+	L+	H+	L+
Biochar addition to soil	0.03–6.6	Up to 3200	L+	M+	H−	L−
Carbon storage in seabed	0.5–2.0	nd	L+	nd	Nd	nd
Enhanced mineral weathering	0.5–4.0	nd	L−	L−	L+	L−
Enhanced urban food systems	nd	nd	M+	nd	H+	nd
Fire management	0.48–8.1	> 5.8	L+	L+	M+	H+
Improved and sustainable forest management	0.4–2.1	> 25	H+	H+	M+	M+
Improved cropland management	1.4–2.3	> 25	M+	L+	H+	L+
Improved grazing land management	1.4–1.8	1–25	M+	L+	H+	L+
Improved livestock management	0.2–2.4	1–25	M+	L+	H+	nd
Increased soil organic carbon content	0.4–8.6	Up to 3200	M+	H+	H+	M+
Integrated coastal zone management	5	200–3000	M+	M+	Variable	Variable
Integrated water resource management	0.1–0.72	250	M+	H+	H+	M+
Management for biodiversity and ecosystem services	0.4–7.6	30–1600	H+	H+	H+	H+
Management of coastal and marine ecosystems	0.5–1.38	nd	M/H+	nd	H+	M/H+
Management of food supply chains	nd	> 100	M+	L+	M+	L+
Management of invasive species/encroachment	nd	nd	H+	L+	nd	L+
More sustainable ocean fisheries, aquaculture and dietary shifts	0.30–1.47	nd	M/H+	H+	H+	H+
Nature conservation	0.9	Likely many millions	H+	H+	nd	nd^a^
Nature‐based ILK	d	nd	H+	H+	H+	H+
Reduced conversion of grassland to cropland	0.03–0.7	nd	H+	L+	M−	nd
Reduced deforestation and degradation	0.4–5.8	1–25	H+	H+	L+	L+
Reduced post‐harvest food losses	4.5	320–400	M/H+	L+	H+	H+
Reduced soil erosion	Source of 1.36–3.67 to sink of 0.44–3.67	Up to 3200	L+	M+	H+	M+
Reforestation and forest restoration	1.5–10.1	> 25	H+	Variable	M−	L+
Restoration and reduced conversion of peatlands	0.6–2.0	nd	H+	H+	L+	L+
Rewilding	0.3–10.1	> 25	H+	H+	L−	H+
Sustainable intensification	> 13	> 163	M/L+	Variable	H+	H+

*Note:* H, M and L indicate high, medium and low impact. Positive impacts are shown in shades of blue and demoted with +, negative impacts are shown in shades of orange and denoted with − (light colours = lower impact; darker colours = higher impact). Variable impacts are shown by grey shading. Insufficient data are shown as ‘nd’. Sources of data used to compile this table came from Araújo and Alagador (2024), Babiker et al. (2022), Bezner Kerr et al. (2023), Canadell et al. (2021), Rodriguez et al. (2024), Chang et al. (2021), Collins et al. (2024), Daigneault et al. (2022), Foti et al. (2020), Giljum et al. (2022), Gupta and Dube (2018), Gurgel et al. (2024), Herrero et al. (2016), Hisano et al. (2018), Hoegh‐Guldberg and Northrop (2019), Hoegh‐Guldberg et al. (2023), Houghton et al. (2015), IPBES (2019a), Kaluwin and Smith (1997), Koh et al. (2021), Körner et al. (2017), McElwee et al. (2020), McLeod et al. (2011), Moorhouse and Sandom (2015), Mori (2020), Mori et al. (2021), Raj et al. (2021), , Sharma and Birman (2024), Skilleter and Warren (2000), Schmitz et al. (2023), Smith et al. (2019, 2020, 2022), Stentiford et al. (2020), Svenning (2020), UN (2014) and Vohland et al. (2012).

^a^ Note that other chapters in the IPBES Nexus Assessment (IPBES 2024) scored a subset of nature conservation actions and found many to have high positive impacts for health.

**TABLE 7:** Impacts of climate change response options under the Energy System Transition category on biodiversity, water, food system and human health and well‐being.

Response option	Mitigation impact (GtCO2e/yr)	Adaptation impact (Millions of people)	Biodiversity impact	Water impact	Food system impact	Health and well‐being impact
Bioenergy and biofuels	0.43–1.29	Variable (M− to L+)	Variable	Variable	L−	L−
Bioenergy and CCS (BECCS)	0.5–11	L−	M−	M−	H−	L−
Energy demand management	2–3	Millions	L+	L+	L+	L+
Energy storage for low‐carbon grids	1.5–2.3	Billions	Variable	L−	nd	L+
Energy system integration	4–6	Billions	nd	L+	nd	L+
Fossil fuels with CCS	0.27–5	nd	L−	L−	nd	L−
Geothermal energy	0.37–1.11	nd	L−	H−	L+	L−
Hydroelectric power	0.16–0.48	L−	H−	Variable	L−	Variable
Nuclear power	0.44–1.32	nd	L−	L−	nd	Variable
Ocean‐based renewable energy	0.05–1.90	nd	Variable	H+	M+	M+
Resilient power infrastructures/systems	1–2	Hundreds of millions	nd	L+	L+	L+
Solar PV (offshore)	0.17^a^	nd	Variable	H+	M+	M+
Solar PV on land	2.0–7.0	Millions	Variable	M+	Variable	M+
Wind power (offshore)	0.30–3.5	Many millions	Variable	H+	M+	M+
Wind power (onshore)	2.1–5.6	Millions	M−	M+	Variable	Variable

*Note:* H, M and L indicate high, medium and low impact. Positive impacts are shown in shades of blue and demoted with +, negative impacts are shown in shades of orange and denoted with − (light colours = lower impact; darker colours = higher impact). Variable impacts are shown by grey shading. Insufficient data are shown as ‘nd’. Sources of data used to compile this table came from Adair‐Rohani et al. (2013), Aman et al. (2015), Barron‐Gafford et al. (2019), Barthelmie and Pryor (2021), Berga (2016), Bergström et al. (2013), Carbon Trust (2024), Cavalett et al. (2022), Clarke et al. (2022), Cormos et al. (2013), Dai et al. (2015), Dhakal et al. (2022), Dhar et al. (2020), Dholakia (2018), Dorber et al. (2018), Douziech et al. (2016), Dunlap (2018), Englund et al. (2020), Eswara and Ramakrishnarao (2013), Floret et al. (2022) Gracey and Verones (2016), Gibon et al. (2017), GWEC (2020, 2021, 2024), Hallosserie et al. (2019), He et al. (2019), Hertwich et al. (2016), Hoegh‐Guldberg and Northrop (2019), Hooper et al. (2021), IEA (2020), IRENA (2019, 2021), Jacobson (2019), Jain et al. (2021), Jensen (2020), Kharecha and Hansen (2013), Kim and Koo (2016), Kinney et al. (2019), Laranjeiro et al. (2018), Macknick et al. (2012), May et al. (2020), McCombie and Jefferson (2016), McElwee et al. (2020), Morris and Blekkenhorst (2017), Næss et al. (2023), Parati et al. (2010), Popp et al. (2014), Pörtner et al. (2021), Robertson et al. (2017), Roy et al. (2018), Santangeli et al. (2016), Schipper et al. (2022), Skarin et al. (2015), Soltani et al. (2021), Smith et al. (2020), Stefanelli et al. (2019),Tawalbeh et al. (2021), US Government (2013), WHO (2024), World Bank (2024), World Economic Forum (2024), Yang et al. (2020), Ziv et al. (2012) and Zhang et al. (2018).

^a^ Based of 36 GW potential off South America only (IRENA 2021) and using the current global energy mix to calculate the mitigation potential.

**TABLE 8:** Impacts of climate change response options under the Settlement and Infrastructural System Transition category on biodiversity, water, food system and human health and well‐being.

Response option	Mitigation impact (GtCO2e/yr)	Adaptation impact (Millions of people)	Biodiversity impact	Water impact	Food system impact	Health and well‐being impact
Change in construction methods and materials	Up to 8	Millions	nd	L+	nd	M+
Efficient appliances	Up to 9.2	Hundreds of millions	nd	M+	nd	nd
Electromobility	Up to 4.7	Millions	nd	nd	nd	Variable
Energy‐efficient building	Up to 5	Millions	nd	nd	nd	M+
Green mobility	Up to 4.7	Millions	nd	nd	nd	H+
Multi‐hazard early warning systems	nd	393	M+	M+	M+	M+
Sustainable land‐use and urban planning	Up to 3.7	Millions	L+	L+	L+	M+
Urban green/blue infrastructure	0.5–2	6700	L+	L+	L+	H/variable
Urban nature‐based solutions (ecopolis)	0.5–2	6700	L+	M+	M+	Variable
Waste prevention, minimisation and management	0.6–0.8	nd	L+	H+	H+	M+

*Note:* H, M and L indicate high, medium and low impact. Positive impacts are shown in shades of blue and demoted with +, negative impacts are shown in shades of orange and denoted with − (light colours = lower impact; darker colours = higher impact). Variable impacts are shown by grey shading. Insufficient data is shown as ‘nd’. Sources of data used to compile this table came from Ackerman (2000), Banwell et al. (2018), Brooks et al. (2009), Burchart‐Korol and Folęga (2019), Cabeza et al. (2022), CMIC (2024), Downton (2012), Escosteguy et al. (2023), Felton et al. (2010), Frantzeskaki et al. (2019), Gómez‐Sanabria et al. (2022), Green et al. (2014), Hashemi (2016), IEA (2021), IUCN (2024), Koop and van Leeuwen (2017), Kopecká et al. (2024), Lam (1999), Lwasa et al. (2022), O'Grady (2010), Pathak et al. (2022), Reynolds et al. (2020), Rossbach (2024), Sanders and Phillipson (2003), Santos et al. (2021), Sturiale and Scuderi (2019), Tirado et al. (2022), Turnbull et al. (2013), Tzoulas et al. (2007), United Nations (2015), UNDRR (2022, 2023), Wolkinger et al. (2018), WHO (2008), Xue et al. (2021) and Zeng (2024).

**TABLE 9:** Impacts of climate change response options under the Industrial System Transition category on biodiversity, water, food system and human health and well‐being.

Response option	Mitigation impact (GtCO2e/yr)	Adaptation impact (Millions of people)	Biodiversity impact	Water impact	Food system impact	Health and well‐being impact
Carbon‐Neutral Manufacturing	0.37–10	> 5.5	M+	H+	M+	H+
Circular bioeconomy	4.5	Millions	M+	L+	L+	H+
Direct Air Carbon Capture and Storage (DACCS)	5–40	Millions	No effect envisaged	M−	No effect envisaged	No effect envisaged
Direct Air Carbon Capture and Utilisation (DACCU)	1–2	Millions	nd	M−	nd	nd
Eco‐Industrial Parks	nd	Likely many millions	M+	H+	M+	M+
Green innovations (innovations in processes, techniques, systems and products)	> 1	Millions	M+	M+	M+	L+
Green supply chain management	> 1	Millions	M+	L+	M+	L+
Improved materials efficiency	Up to 2	Millions	M−	Variable	nd	nd
Industrial Symbiosis	0.01–1.0	15 million	L+	H+	H+	M+

*Note:* H, M and L indicate high, medium and low impact. Positive impacts are shown in shades of blue and demoted with +, negative impacts are shown in shades of orange and denoted with—(light colours = lower impact; darker colours = higher impact). Variable impacts are shown by grey shading. Variable impacts are shown by grey shading. Insufficient data are shown as ‘nd’. Sources of data used to compile this table came from Babiker et al. (2022), Barecka et al. (2021), Barrios et al. (2020), Benton et al. (2021), Brears (2015), Breyer et al. (2019), Butturi et al. (2019), Castellet‐Viciano et al. (2022), Chen et al. (2022), Elshkaki (2023), Fan et al. (2017), Fraccascia et al. (2021), Geneidy et al. (2023), Guo et al. (2023), Hamam et al. (2023), Herrero et al. (2016), IOSCM (2024), IEAGHG (2022), International Resource Panel (2020), IPCC (2019b, 2022), Karpa (2017), Khan and Johl (2019), Kostyshak et al. (2024), Lazaroiu et al. (2019), Lehtoranta et al. (2011), Leppäkoski et al. (2023), Levin‐Nally and Racionero Gómez (2020), Melnychenko et al. (2022), Persaud and Schillo (2017), Rosado and Kalmykova (2019), Smith et al. (2019), Sokka et al. (2011), Ulusoy et al. (2024), UNEP (2011), Wang et al. (2020), World Economic Forum (2021, 2025).

**TABLE 10:** Impacts of climate change response options under the Social System Transition category on biodiversity, water, food system and human health and well‐being.

Response option	Mitigation impact (GtCO2e/yr)	Adaptation impact (Millions of people)	Biodiversity impact	Water impact	Food system impact	Health and well‐being impact
Behavioural nudges for sustainability	0.7–8.0	Millions	H+	H+	H+	H+
Dietary change (sustainable healthy diets)	0.7–8.0	> 1900	H+	L+	H+	H+
Reduced food waste (consumer and retailer)	0.8–4.5	> 783	M/H+	L+	H+	M+

*Note:* H, M and L indicate high, medium and low impact. Positive impacts are shown in shades of blue and demoted with +, negative impacts are shown in shades of orange and denoted with—(light colours = lower impact; darker colours = higher impact). Variable impacts are shown by grey shading. Sources of data used to compile this table came from Blackford (2021), Falcone and Fiorentino (2024), IPCC (2019b), Ispiryan et al. (2024), Herrero et al. (2016), McElwee et al. (2020), Oh et al. (2024), Reisch (2021), Saunders et al. (2006), Smith et al. (2019, 2020, 2022), Tirado et al. (2022), UNEP (2017), UNFCCC (2024), UNSCN (2017), Veríssimo et al. (2024), Williamson et al. (2018) and Winterstein (2022).

The 11 (35%) response options under the Land, Ocean and Ecosystems Transition category that can be scored for all elements and were positive for all of climate change mitigation and adaptation, biodiversity, water, food system and human health and well‐being are increased soil organic carbon content, improved and sustainable forest management, agroecology, reduced deforestation and degradation, fire management, improved cropland management, improved grazing land management, agroforestry, integrated water management, reduced post‐harvest food losses and management for biodiversity and ecosystem services (Table 6).

A further 12 (39%) response options have insufficient data for at least one element but otherwise report only positive impacts. These are carbon storage in the seabed, reduced conversion and restoration of peatlands, management of coastal and marine ecosystems, more sustainable ocean fisheries, aquaculture and dietary shifts, management of food supply chains, enhanced urban food systems, improved livestock management, agricultural diversification, management of invasive species/encroachment, nature conservation and nature‐based Indigenous and local knowledge (ILK).

There are six (19%) response options that have a potential negative impact on at least one of the elements. These are (1) afforestation, with potentially negative impacts on biodiversity if monoculture trees replace, for example, more diverse grasslands (Smith et al. 2022), and on the food system, due to land taken out of production (Smith et al. 2020); (2) biochar addition to soil, with potentially negative impacts on the food system if large areas of land used to produce food are required for biochar feedstock, though the impact could be positive if the biochar is produced from crop residues (Smith 2016; Smith et al. 2020), and human health and well‐being due to air pollutants from biomass pyrolysis (Li 2024); (3) enhanced mineral weathering, with potential negative impacts on biodiversity from increased mining (Giljum et al. 2022), water used in mining and rock grinding (Smith et al. 2016; Eufrasio et al. 2022) and on human health and well‐being from increased mining (Giljum et al. 2022); (4) reduced conversion of grassland to cropland, which could impact negatively on food security (Smith et al. 2020); (5) reforestation and forest restoration, with potentially negative impacts on the food system if large areas of land are converted for tree planting—though increased forest cover in forest biomes can improve yields in cases where it protects/enhances water cycling and precipitation, and reduces heat stress in areas adjacent to agricultural areas (Smith et al. 2020) and (6) rewilding, which can take land out of production for food provision (Smith et al. 2020).

There are five (16%) response options with variable impacts (both positive and negative impacts reported in the literature depending on context) on at least one element. Both afforestation and reforestation and forest restoration have a variable impact on water depending on the tree species and the vegetation that they replace, with tree species having a greater water use than low stature vegetation (Smith et al. 2016). Sustainable intensification will likely have a positive impact on water, but if relying on additional irrigation, could contribute to a larger water footprint (Muleke et al. 2023). Integrated coastal zone management could have variable impacts on the food system, with some forms of restoration allowing coastal food production, but other forms adversely affecting coastal fisheries (Munang et al. 2014), and human health and well‐being having positive effects on health if protecting against coastal flooding (Munang et al. 2014), but potentially negative impacts if encouraging water‐borne disease vectors (Dale and Connelly 2012).

### 3.2 Energy System Transition Response Options

While all of the Energy System Transition response options contribute to climate change mitigation (potentials to 2050) and many also contribute to adaptation (other than hydroelectric power and BECCS which can have negative impacts on adaptation), only one (7%) of the 15 (demand‐side mitigation) contributes positively to each of the nexus elements of biodiversity, water, food system and human health and well‐being. Two others (13%) have no documented or variable or negative effects (energy system integration and resilient power infrastructures/systems), but there was insufficient data to assess the impact on at least one element. Seven (47%) response options have documented negative impacts on at least one element, whereas nine (60%) have variable impacts (both positive and negative impacts reported in the literature depending on context) on at least one of biodiversity, water, food system and human health and well‐being (Table 7).

The eight (53%) response options that have documented negative impacts on at least one of biodiversity, water, food system and human health and well‐being are onshore wind power, hydroelectric power, nuclear power, geothermal energy, bioenergy and biofuels, bioenergy with carbon capture and storage (CCS), fossil fuels with CCS and energy storage for low carbon grids.

Onshore and offshore wind power can have a negative impact on biodiversity if turbines and other infrastructure associated with large‐scale wind farms impinge upon areas important for biodiversity conservation, for example, protected areas (e.g., Santangeli et al. 2016; Lloret et al. 2022; Galparsoro et al. 2022) or through the mining of rare earth metals used in turbines (McCombie and Jefferson 2016; Valero et al. 2018).

The negative impacts on biodiversity of flooding areas to create dams for hydroelectric power are well documented (Clarke et al. 2022; Dorber et al. 2018; Gracey and Verones 2016; Hallosserie et al. 2019; Hertwich et al. 2016; Pörtner et al. 2021; Roy et al. 2018; Ziv et al. 2012), and such flooding for dam creation can also affect local food systems (Zhang et al. 2018; Ziv et al. 2012).

Nuclear power can have negative impacts on biodiversity and on water through the mining of ores for fuel and via nuclear waste disposal (Clarke et al. 2022; McCombie and Jefferson 2016; Roy et al. 2018).

Geothermal energy can have large negative impacts on water quality through groundwater contamination (Clarke et al. 2022; Hertwich et al. 2016; Soltani et al. 2021) and can also have a negative impact on biodiversity through habitat loss and human health and well‐being through air pollution (Clarke et al. 2022; Hallosserie et al. 2019; Hertwich et al. 2016).

Bioenergy and biofuels can have a negative impact on food production if large tranches of land that would otherwise be used to produce food are put aside for energy production (Englund et al. 2020; McElwee et al. 2020; Næss et al. 2023; Popp et al. 2014; Roy et al. 2018) and can have a negative impact on human health and well‐being through air and water pollution from the combustion of biomass and biofuels and use of fertilizers (Clarke et al. 2022; Gibon et al. 2017; McElwee et al. 2020; Roy et al. 2018). When combined with CCS (i.e., BECCS), some of the negative impacts from bioenergy are ameliorated by the CCS; for example, air pollution can be reduced, while others can be exacerbated by the CCS; for example, the water footprint increases significantly with CCS (Smith et al. 2016). When assessing BECCS specifically, the potential negative impact on the food system is high (McElwee et al. 2020; Smith et al. 2020), with potential negative impacts also on human health and well‐being (Gibon et al. 2017; McElwee et al. 2020; Roy et al. 2018), water (Clarke et al. 2022; Englund et al. 2020; McElwee et al. 2020; Roy et al. 2018) and biodiversity (Clarke et al. 2022; Gibon et al. 2017; Hallosserie et al. 2019; McElwee et al. 2020; Robertson et al. 2017; Roy et al. 2018; Santangeli et al. 2016).

Though less damaging than unabated fossil fuel use (Gibon et al. 2017), fossil fuels with CCS impact negatively on biodiversity through damage to ecosystem quality (Gibon et al. 2017; Hertwich et al. 2016), water, through consumption of water for CCS (Cormos et al. 2013; Yang et al. 2020) and human health and well‐being, mainly via reductions in ecosystem quality and air pollution (Cavalett et al. 2022; Clarke et al. 2022; Gibon et al. 2017; Hertwich et al. 2016; Jacobson 2019).

Energy storage for low‐carbon grids can have a negative impact on water, if, for example, implemented through pumped hydroelectric storage; but there are many energy storage technologies, including batteries, liquid air and thermal energy storage, flywheels and supercapacitors, redox flow batteries, hydrogen and reversible hydrogen fuel cells and power to fuels, each with varying impact on biodiversity and water (Clarke et al. 2022).

The nine (60%) response options that have variable impacts (both positive and negative impacts reported in the literature depended on context) on at least one of biodiversity, water, food system and human health and well‐being are solar PV on land, solar PV offshore, onshore and offshore wind power, ocean‐based renewable energy, hydroelectric power, nuclear power, bioenergy and biofuels and energy for low‐carbon grids.

Solar PV on land can have positive or negative impacts on biodiversity and the food system; negative impacts may occur if sited on land rich in biodiversity, though biodiversity can be preserved beneath arrays of solar panels providing a positive impact (Aman et al. 2015; Clarke et al. 2022; Dhar et al. 2020; Douziech et al. 2016; Hallosserie et al. 2019; Hertwich et al. 2016; Pörtner et al. 2021; Santangeli et al. 2016). Negative impacts of solar PV on land for food occur if solar farms occupy land used for food production, but if planned carefully, grazing or crop production can occur in synergy with electricity production, through so‐called agro‐voltaics (Barron‐Gafford et al. 2019; Jain et al. 2021; Eswara and Ramakrishnarao 2013; He et al. 2019; Roy et al. 2018; Tawalbeh et al. 2021). Offshore solar PV can have positive or negative impacts on biodiversity. Negative impacts may occur for floating PV, particularly for corals and seagrasses, though there can be co‐benefits from artificial reef effects and through multi‐use platforms (Hooper et al. 2021).

Onshore wind power can have variable impacts on food systems (He et al. 2019, Morris and Blekkenhorst 2017) and on human health and well‐being (Clarke et al. 2022; Dunlap 2018; Roy et al. 2018). Positive impacts on food production may occur since wind energy enhances drought resilience and groundwater sustainability (He et al. 2019), whereas negative impacts may occur due to altering the agricultural land base (Morris and Blekkenhorst 2017), though food production usually continues beneath turbines. In terms of human health and well‐being, Dunlap (2018) reported negative impacts of wind farms on Indigenous communities in the coastal isthmus of the Tehuantepec region of Oaxaca, Mexico, due to land losses and environmental impacts of construction. In other places like the US and Canada, Indigenous and tribal communities have been able to develop wind energy successfully with multiple social benefits, particularly, when there is Native ownership of the system, resulting in a reduction of pollution harms (Konkel 2013; Stefanelli et al. 2018; Grosse and Mark 2023); however, there are also reported negative impacts, like effects on sacred sites (Grosse and Mark 2023). Offshore wind power can have variable impacts on biodiversity, with offshore turbines and associated structures potentially impacting migratory birds, seabirds, marine mammals, reptiles seabed habitats, sedimentary processes (Hooper et al. 2017; Clarke et al. 2022, Dannheim et al. 2020, Lloret et al. 2022, Galparsoro et al. 2022) and even water mixing (Dorrell et al. 2022), but as with offshore PV, there can be co‐benefits from artificial reef effects, exclusion of trawling from fishing, acting as other effective area‐based conservation measures (with spillover effects) and through multi‐use platforms (Hammar et al. 2016, Degraer et al. 2020, Hooper et al. 2021, Ingle et al. 2022, Huang, Afero, et al. 2022). Other ocean‐based renewable energies have variable impacts on biodiversity. While some forms (such as offshore wind and PV) can have biodiversity benefits and adverse impacts, other forms, such as algal biomass for BECCS and tidal power, have context‐specific impacts on biodiversity (Douziech et al. 2016; Hallosserie et al. 2019; Hoegh‐Guldberg and Northrop 2019; Hooper et al. 2021; Kim and Koo 2016).

The variable impacts of hydroelectric power on water arise from the negative impacts of dam creation for hydropower on water quality; the potential positive impact of controlled downstream water flow (Clarke et al. 2022; Hallosserie et al. 2019; Hertwich et al. 2016; Roy et al. 2018).

The impacts of bioenergy and biofuels are variable for biodiversity and water. The biodiversity impact of energy crops can be positive, for example, when perennial energy crops replace monoculture food crops (Lovett et al. 2015), or negative when implemented at large scale through impinging on land used for nature conservation (Clarke et al. 2022; Gibon et al. 2017; Hallosserie et al. 2019; Santangeli et al. 2016; Roy et al. 2018; Robertson et al. 2017). For water, the negative impact of bioenergy results from the higher water use of perennial energy crops compared to short‐stature annual food crops (Smith et al. 2016), whereas a positive impact on water (Roy et al. 2018; Clarke et al. 2022) can result from energy crops that require less fertilisation than food crops and that prevent soil erosion, thereby improving water quality (Englund et al. 2020; Smith et al. 2016).

The variable impact of energy storage for low‐carbon grids on biodiversity arises due to the large number of technologies that fall under this umbrella. Pumped hydroelectric energy storage, for example, has a large water footprint, whereas the overall benefits of allowing a more effective switch away from fossil fuels that is afforded by energy storage for low‐carbon grids undoubtedly benefit biodiversity (Clarke et al. 2022).

### 3.3 Settlement and Infrastructural System Transition Response Options

Four of the ten (40%) Settlement and Infrastructural System Transition response options—waste prevention, minimisation and management, sustainable land‐use and urban planning, disaster risk reduction and management—contribute positively to each of biodiversity, water, food system and human health and well‐being. Five (50%) response options lack sufficient data to assess the impact on at least one element, with four of these five options, namely efficient appliances, change in construction methods and materials, energy efficient buildings and green mobility, having a positive impact (and no negative impacts) for the one or two elements that can be assessed (Table 8).

Three (30%) response options have variable impacts (both positive and negative impacts reported in the literature depending on context) on human health and well‐being: urban nature‐based solutions (Downton 2012), electromobility and urban green/blue infrastructure. Urban green/blue infrastructure and urban nature‐based solutions can have positive effects on physical and mental health and well‐being (Tirado et al. 2022; Tzoulas et al. 2007) but can sometimes present health risks; for example, when rewilding or rewetting urban wetland areas increases the risk of insect vector‐borne diseases (Dale and Connelly 2012). Electromobility will generally enhance human health by reducing air pollution in urban areas (e.g., Burchart‐Korol and Folęga 2019), but concerns have been raised about negative well‐being impacts from the mining of rare earth metals used in electrified transport (Escosteguy et al. 2023).

### 3.4 Industrial System Transition Response Options

Of the nine Industrial System Transition response options assessed, six (67%) contribute positively to each of biodiversity, water, food system and human health and well‐being. Three (33%) have potential negative impacts on one element, one (11%) has variable impacts (both positive and negative impacts reported in the literature depending on context) on one element, and there is insufficient data to assess the impact of three (33%) response options on at least one element (Table 9).

The six (67%) Industrial System Transition response options that contribute positively to each of biodiversity, water, food system and human health and well‐being are sustainable (including circular) bioeconomy (Yang et al. 2020; Sauvé et al. 2021; Venkatesh 2022; Trigo et al. 2023; Miranda et al. 2020; Gomez San Juan et al. 2022), green innovations (innovations in processes, techniques, systems and products), green supply chain management (Barrios et al. 2020; Khan and Johl 2019; Lazaroiu et al. 2019; Persaud and Schillo 2017; Karpa 2017), eco‐industrial parks, carbon‐neutral manufacturing and industrial symbiosis.

The three (33%) Industrial System Transition response options that have potential negative impacts on one element are improved materials efficiency, Direct Air Carbon Capture and Storage (DACCS) and Direct Air Carbon Capture and Utilisation (DACCU). Improved materials efficiency, if implemented via technologies that require additional mined materials, may have negative impacts on biodiversity (Levin‐Nally and Racionero Gómez 2020). The CCS component of DACCS and DACCU has a large water footprint (Smith et al. 2016).

The Industrial System Transition response option that has variable impacts (both positive and negative impacts reported in the literature depending on context) on one element is improved materials efficiency. There is a variable impact on water from improved materials efficiency, since the production, use and waste management of materials require energy and water (Elshkaki 2023); thus, depending on the material transition, it may have either a positive or negative impact on water use.

### 3.5 Social System Transition Response Options

Only three Social System Transition response options were considered. Dietary change towards sustainable healthy diets, reduced food waste (consumer and retailer) and behavioural nudges for sustainability all (100%) contribute positively to each of biodiversity, water, food system and human health and well‐being (Table 10).

## 4 Synthesis Across the System Transitions

**FIGURE 1:**
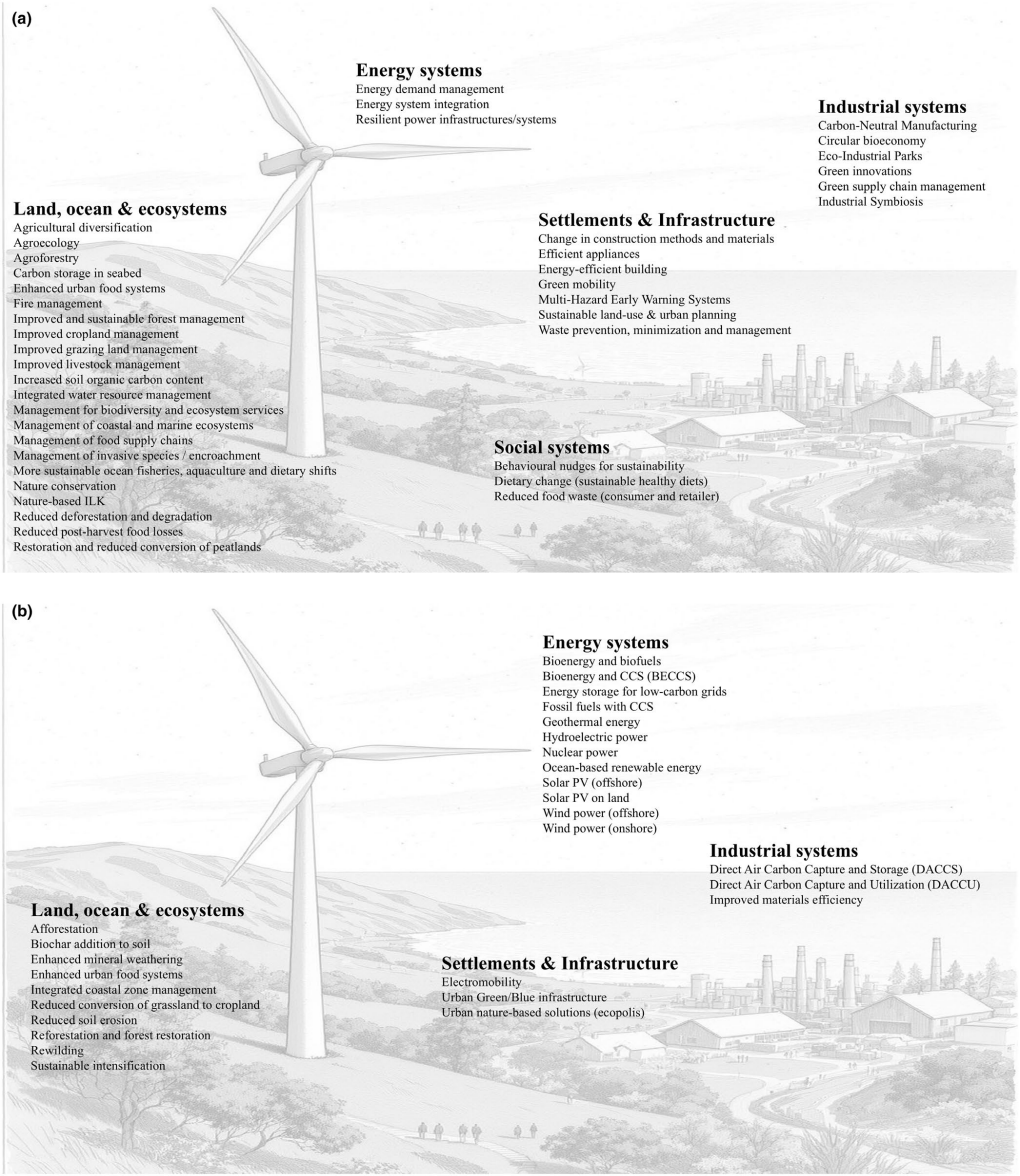
Synergistic response options—that have entirely positive or no negative or variable impacts on any of the nexus elements (a) and trade‐off response options—that show potential negative or variable impacts for at least one nexus element (b).

Across the five system transitions, 41 (59%) of the 69 response options have no negative or variable impacts on any of the nexus elements (Figure 1a), suggesting that they are low‐risk options for addressing climate change, whereas 28 (41%) show potential negative or variable impacts for at least one nexus element (Figure 1b), meaning that they need to be implemented in ways which minimise adverse outcomes.

## 5 Discussion

Climate change threatens all nexus elements: biodiversity, water, food and human health and well‐being. Effective, rapid, sustained mitigation and accelerated implementation of mitigation and adaptation actions have the potential to benefit all nexus elements while minimising trade‐offs and fostering synergistic outcomes. Rapid transitions in energy use and production, land and marine ecosystem management, urban areas, industrial activities, and society's behaviours, attitudes and values are crucial for enabling climate change mitigation, adaptation and sustainable development. These transitions require interdisciplinary research, policy support and societal engagement. Enabling transformative change towards a climate‐resilient future requires enhancing synergies and managing trade‐offs among climate change adaptation, mitigation and other nexus elements.

Transitions in terrestrial and marine ecosystem management, through response options such as sustainable intensification, agroecology, forest‐based practices to address climate change, peatland/wetland conservation, restoration of blue carbon ecosystems (seagrasses, saltmarshes and mangroves) and integrated multi‐trophic aquaculture, can also support climate change mitigation and adaptation while benefiting the other nexus elements. For the Land, Ocean and Ecosystems Transition response options, while some can have variable or even potential negative impacts on one or more of biodiversity, water, food systems and human health and well‐being, the majority show a range of co‐benefits across all elements, providing policymakers with many options to tackle multiple challenges together through land and ocean ecosystem interventions (Figure 1). Options that avoid competing with land used for food production tend to lead to better system‐wide outcomes. This is in line with the findings of Smith et al. (2020) and McElwee et al. (2020) who found many synergies for climate change mitigation and adaptation, food security and halting land degradation and desertification among land‐based response options, and Pörtner et al. (2021) and Smith et al. (2022) who found synergies for land and ocean ecosystem‐based response options for climate change mitigation and adaptation and biodiversity.

For Energy System Transition response options, rapid transitions in energy use and production include the replacement of energy generated from fossil fuels with the rapid expansion of renewable energy, such as wind power and solar photovoltaics, improvements in efficiency and reductions in consumption. Shifting away from fossil fuels and deploying renewable energy is essential for abating the extremely negative impacts of climate change on all the other nexus elements, which include droughts, floods, fires, heat stress, sea‐level rise, etc. While only three out of 15 (20%) response options in this category were scored as having positive impacts across all nexus elements, this scoring may underestimate their systemic benefits. These technologies play a critical role in reducing emissions and climate change impacts, which in turn supports improvements in biodiversity, food and water security and health. Apparent negative or variable impacts of energy system transitions (Figure 1b) should be interpreted in the context of the fossil fuels they replace. Many infrastructure‐related impacts associated with renewables would also occur under fossil fuel‐based systems. For example, Wang et al. (2015) reported that wind turbines in the USA were responsible for 140 thousand bird deaths; yet, transmission lines caused an estimated 174 million bird fatalities over the same period. Thus, impacts must be assessed relative to the counterfactual of incumbent fossil fuel technologies and infrastructures. Fossil fuels contribute to climate change and air pollution, both of which have widespread adverse effects on biodiversity, ecosystems and human health. Transitioning to cleaner, more renewable energy sources would reduce these impacts and offer significant co‐benefits across the nexus. Focussing only on direct impacts of the implementation of a response option (as in this study), rather than focusing on the indirect, system‐wide impacts of a transition away from fossil fuels towards more renewable energy, is a limitation of the approach taken here. Integrated Assessment Models are needed to examine such system‐wide impacts. The context specificity of the impacts is clear from the many categories for which variable impacts were found. For example, solar PV on land could compete with land for food and biodiversity conservation, but these risks would be negated if the solar panels are sited on built infrastructure, such as rooftops. Given the range of potential negative impacts, attention is required to systemic change, going beyond focussing only on technologies, and instead also focussing on reducing energy consumption.

For Settlement and Infrastructural System Transition response options, while there is a lack of data for a number of options, none were found to have solely negative impacts on any element, with three having variable impacts for one element, and four options having positive impacts on each of biodiversity, water, food system and human health and well‐being (Figure 1). This suggests that most response options to tackle climate change in the Settlement and Infrastructural System Transition category pose a low risk to the other nexus elements.

For Industrial System Transition response options, three have either negative or variable impacts on one or two elements (Figure 1b), whereas two contribute positively to each of biodiversity, water, food system and human health and well‐being (Figure 1a). This suggests that some response options to tackle climate change in the Industrial System Transition category pose a low risk to other elements, whereas others need to be managed carefully to avoid adverse impacts.

For Social System Transition response options, the three selected, both reducing food loss and waste and dietary change towards sustainable healthy diets, are low‐risk options for addressing climate change, while also providing benefits for biodiversity, water, the food system and human health and well‐being (Figure 1a). Only three options were examined here, but given the nexus synergies afforded by Social System Transition response options, future research should focus on social, institutional, governance and value‐related dimensions of societal system transitions. Behaviour change (such as reducing demand) has been shown to be critical for addressing many global challenges facing humanity (Hertwig et al. 2025).

Studies such as this one, and previous studies taking a similar approach such as Smith et al. (2020), McElwee et al. (2020), Pörtner et al. (2021) and Smith et al. (2022), are limited by the literature that can be assessed, limitations in the methods of scoring, the scale at which response options are assessed and the way various studies showing different outcomes are summarised. As Smith et al. (2022) noted, implementation is key; just about any response option could be scored ‘it depends on how it is implemented’. The aim of this assessment was to provide the best scoring possible for each option/nexus element combination. It would be possible to do a full systematic review of each option/nexus element combination (69 × 6 = 414), which would provide a series of more comprehensive assessments than was possible here. In this respect, this study could also be used as a map for future systematic reviews and further research.

The options that contribute positively across all nexus elements have higher transformative change potential and bridge inequity gaps because they co‐deliver solutions to a range of global challenges. For example, agroecology has documented potential to not only improve all nexus elements but also to improve equity in farming communities that practice its principles (Bezner Kerr et al. 2023). Some response options enable the successful implementation of others, highlighting that bundling and sequencing of response options can help deliver transformative change at lower costs compared with the sum of options deployed individually. For example, the reduced pressure on land arising from shifting to sustainable healthy diets in overconsuming regions (Hayek et al. 2021) and/or through sustainable intensification (Pretty et al. 2018) can enable more sustainable forms of farming such as agroecology (Bezner Kerr et al. 2023). This can free up land for other land‐based climate change response options, such as reforestation, forest‐based practices to address climate change, wetland and peatland restoration and conservation (Hayek et al. 2021) or land‐based renewable energy (Lamb et al. 2016). Renewable energy options provide high climate change mitigation potential, but like any infrastructure project, they can have unavoidable negative effects on nexus elements. For example, offshore wind power can impact marine ecosystem structure and functioning, and associated fisheries, if poorly planned (Hooper et al., 2017; Clarke et al. 2022). Integrating renewable energy production with energy efficiency measures, or within existing agricultural, urban and marine systems, reduces such risks (Smith et al. 2020). Taking a holistic approach ensures sustainability and mitigates potential harm.

Prioritisation of policies that address the interconnected challenges of climate change, biodiversity loss, land degradation, pollution, food insecurity, access to clean water and energy for all and sustainable development delivers more effective climate solutions. Key considerations include integrating food systems and access to sustainable healthy diets in climate action plans, strengthening land use governance, optimising finance and building capacity for renewable energy production and addressing the biodiversity and climate crises and engaging in both formal and informal governance interactions (Singh et al. 2024).

Failures to account for interactions will have serious consequences in the climate sector. Response options designed solely for climate change mitigation, such as large‐scale afforestation with non‐native species in ecologically incongruent regions, tend to have more pronounced negative impacts (Hua et al. 2022). These may lead to resource competition or ecological imbalances. In contrast, strategies that take into account the impacts across other elements can maximise synergies. For instance, a shift to healthy sustainable diets can reduce GHG emissions (Bajželj et al. 2014), alleviate pressure on land and water resources (Hayek et al. 2021) and lower public health costs associated with poor nutrition (Scarborough et al. 2011), thereby acting as an enabler for other response options to be implemented successfully. When conflicts arise across the elements, tailored management strategies are needed to mitigate adverse effects.

Inclusive and participatory governance is essential for ensuring that response options equitably benefit biodiversity, water, food and health systems. The involvement of diverse actors, particularly Indigenous Peoples and local communities (IPLCs), is crucial in shaping climate policies that align with local realities and ecological knowledge (McElwee et al. 2020). However, mainstream governance structures and plans often marginalise these groups, overlooking their holistic approaches to managing ecosystems (Khan et al. 2024). IPLCs conceptualise the interlinkages among biodiversity, water and food security as an integrated whole rather than as separate policy domains; yet their governance systems remain underrepresented in decision‐making processes (IPBES 2024). Recognising rights‐based governance, such as securing land tenure and implementing Free, Prior and Informed Consent (FPIC) protocols, can enhance their leadership in climate adaptation and biodiversity conservation (Denton et al. 2022).

Despite increasing commitments to participatory decision‐making, structural and institutional barriers persist. Power asymmetries between national governments and IPLCs often lead to policy capture by dominant actors, limiting the influence of marginalised communities in climate governance (Kelemen et al. 2022). Additionally, financial and technical constraints hinder the ability of local actors to scale community‐driven conservation and adaptation efforts (Denton et al. 2022). Addressing these barriers requires the establishment of inclusive policy frameworks and multi‐stakeholder governance structures that recognise IPLCs’ knowledge systems and enable their meaningful participation in decision‐making (IPBES 2024). Furthermore, legal recognition of customary governance structures can facilitate a shift towards adaptive governance frameworks that integrate diverse knowledge systems and ensure long‐term sustainability (McElwee et al. 2020).

Strengthening participatory governance mechanisms not only enhances equity but also fosters policy innovation and resilience. By embedding co‐production of knowledge within global frameworks that address climate change, such as the Kunming‐Montreal Global Biodiversity Framework and the 2030 Agenda for Sustainable Development, governance systems can bridge the science‐policy‐practice divide, ensuring that climate interventions empower rather than impose externally designed solutions (IPBES 2019b). Integrating inclusive governance principles into climate policy will be critical for delivering effective, equitable and sustainable interventions in the long term. Some of the negative reported impacts can be systematically addressed in the applicable local contexts for effective nexus solutions when principles of equity (e.g., procedural, distributional, recognitional) are embedded in solutions (IPBES 2024). The rapid transformation, through systems transitions of energy, land‐use, urban planning, industrial activities and societal behaviours is necessary for holistic climate change mitigation, adaptation and sustainable development. Achieving this systemic shift necessitates harmonising various enabling conditions—financial, social, economic, institutional and political. The governance of these sectors is influenced not just by formal institutions but also by a plethora of actors and networks, both of which can either facilitate or obstruct effective solutions. Implementing individual, bundles or sequences of response options is enabled by good governance. Consideration of ethics, values and worldviews is fundamental to such governance as it dictates the inclusivity and effectiveness of climate strategies (Singh et al. 2024).

In light of the benefits of implementing climate change response options within an integrated, inclusive, nexus framework, most (59%) of the response options assessed here have entirely positive effects, or at least no negative effects, across all nexus elements and can be considered synergistic, low‐risk, immediately actionable options (Figure 1a). The remaining (41%) of the response options show either negative impacts or variable impacts on at least one nexus element (Figure 1b). But even for these response options, the potential negative/variable impacts and trade‐offs do not mean they are not actionable—rather, that care must be taken in their implementation to ensure that (a) where impacts are variable, implementation ensures positive outcomes and (b) where one or more nexus elements could be negatively impacted, implementation needs to minimise trade‐offs and maximise synergies. In the literature consulted in this review, there are many case studies showing that response options can be implemented in ways that harness nexus benefits; many excellent examples are given in Singh et al. (2024) and IPBES (2024).

When strategically aligned with nexus elements, integrating diverse options for climate actions presents an unparalleled opportunity to drive transformative change. Through coordinated efforts, inclusive governance and the empowerment of IPLCs, resilience, equity and sustainability can be fostered across all nexus dimensions. By embracing holistic and innovative approaches, the global community can effectively navigate the complexities of climate action, ensuring a balanced and equitable future for all (Singh et al. 2024).

## 6 Conclusions

The findings of this study, which formed part of Chapter 5.5 of the IPBES Nexus Assessment, reinforce the urgent need for integrated climate action that simultaneously advances biodiversity conservation, water security, food system resilience and human health and well‐being. Climate change interventions can generate substantial co‐benefits across these elements, particularly through nature‐based solutions, sustainable land and marine management and systemic energy transitions. However, these interventions may also present some trade‐offs that require careful governance, policy alignment and adaptive management to ensure long‐term sustainability.

Land, ocean and ecosystem‐based response options—such as agroecology, sustainable forest management, reduced deforestation and integrated water resource management—demonstrate strong synergies between climate mitigation, adaptation and broader environmental and social benefits. Expanding these interventions can support biodiversity conservation while enhancing food production and water availability. However, interventions such as large‐scale afforestation, bioenergy expansion and intensive reforestation can undermine biodiversity and food security if not strategically designed and implemented. Similarly, while critical for decarbonisation, energy system transitions must consider the ecological and social costs associated with large‐scale infrastructure development, resource extraction and land‐use change. The negative impacts from energy system transitions like land‐use change, mining and water use can be minimised or avoided with proper planning and governance; for example, through appropriate energy mix planning and siting that optimises across the nexus elements.

Social and behavioural transitions play a crucial role in complementing technological and ecological responses. Dietary shifts towards sustainable and healthy food systems, reducing food waste and behavioural nudges for sustainability offer high‐impact, low‐regret solutions that contribute to both mitigation and adaptation goals. These strategies require robust institutional support, economic incentives and public engagement to drive large‐scale adoption. Strengthening governance frameworks to integrate climate, biodiversity and human development policies is essential for achieving just and sustainable outcomes. Effective governance must prioritise polycentric decision‐making, participatory approaches and financial mechanisms that enable just transitions while ensuring equity and resilience in vulnerable communities.

This assessment highlights the need for transformative climate action that embraces cross‐sectoral synergies and minimises trade‐offs. A shift towards integrated, ecosystem‐based and socially inclusive approaches is necessary to build long‐term resilience. Future research should focus on refining multi‐criteria assessment frameworks to evaluate response options based on their sustainability, feasibility, cost‐effectiveness and long‐term resilience outcomes. Strengthening empirical evidence through case studies and adaptive learning mechanisms will be essential for informing decision‐making processes to learn from what works and understand how implementation and context matter. Climate change mitigation and adaptation efforts must be designed to maximise co‐benefits across interconnected nexus elements, ensuring that responses do not exacerbate existing vulnerabilities but contribute to a just and resilient future.

## Author Contributions

**Pete Smith:** writing – original draft, writing – review and editing. **Pramod K. Singh:** writing – review and editing. **Vedant P. Ballal:** writing – review and editing. **Francesco Cherubini:** writing – review and editing. **Julio Díaz‐José:** writing – review and editing. **Helena Duchková:** writing – review and editing. **Himangana Gupta:** writing – review and editing. **Masakazu Hori:** writing – review and editing. **Akihiko Ito:** writing – review and editing. **Shabana Khan:** writing – review and editing. **Marcos Llope:** writing – review and editing. **Maria Cristina Tirado:** writing – review and editing. **Luara Tourinho:** writing – review and editing. **Mariana M. Vale:** writing – review and editing. **Xiyan Xu:** writing – review and editing. **Harpalsinh Chudasama:** writing – review and editing. **Siri H. Eriksen:** writing – review and editing. **Daniel Mason‐D'Croz:** writing – review and editing. **Sui Chian Phang:** writing – review and editing. **Yash Srivastava:** writing – review and editing. **Tiff L. van Huysen:** writing – review and editing. **Taylor Ricketts:** writing – review and editing. **Mario Herrero:** writing – review and editing. **Paula A. Harrison:** writing – review and editing. **Pamela D. McElwee:** writing – review and editing.

## Conflicts of Interest

The authors declare no conflicts of interest.

## Supporting information


**Data S1:** Supporting Information.

## Data Availability

Data used to compile the tables and associated references are archived as part of the IPBES Nexus Assessment, Chapter 5.5, available at: https://doi.org/10.5281/zenodo.13931518.
