# Supplementary material for: Impacts of Climate Change Interventions on Biodiversity, Water, the Food System and Human Health and Well‐Being
Source: Glob Chang Biol. 2025 Sep 1;31(9):e70444. doi: 10.1111/gcb.70444 (PMC12400271; doi:10.1111/gcb.70444)
Supplement: Supplementary file 1 — Data S1: Supporting Information. [file GCB-31-e70444-s001.docx]

**Supplementary material - criteria for scoring of response options by the authors from data gather from the literature review**

In the sections below, the colour coding of the text corresponds to the colour coding of the cells in tables 6-10 (for ease of visually distinguished direction and extent of positive of negative impact). Shades of blue indicate a positive impact; shades of orange indicate a negative impact - with darker shades indicating larger impact.

1. **Impact of the response option on climate change**

For climate change mitigation, the (realistic technical) GHG emissions reduction/carbon sequestration potential (gigatonnes carbon dioxide equivalent per year; GtCO_2_e/year) of each response option was scored by the authors, as follows:

- High+ (H+) = > 3 GtCO_2_e/year (reduced emissions/positive mitigation potential)
- Medium+ (M+) = 0.3-3 GtCO_2_e/year (reduced emissions/positive mitigation potential)
- Low+ (L+) = positive but < 0.3 GtCO_2_e/year (reduced emissions/positive mitigation potential)
- 0 = no impact on mitigation
- Low- (L-) = up to 0.3 GtCO_2_e/year (increased emissions/negative mitigation potential)
- Medium- (M-) = 0.3-3 GtCO_2_e/year (increased emissions/negative mitigation potential)
- High- (H-) = >3 GtCO_2_e/year (increased emissions/negative mitigation potential)

For climate change adaptation, the number of people benefiting from a response option (millions of people), was scored as follows:

- High+ (H+) = positively impacts more than 25 million people
- Medium+ (M+) = positively impacts 1-25 million people
- Low+ (L+) = positively impacts up to 1 million people
- 0 = no impact on adaptation
- Low- (L-) = negatively impacts up to 1 million people
- Medium- (M-) = negatively impacts 1-25 million people
- High- (H-) = negatively impacts more than 25 million people

1. **Impact of the response option on biodiversity**

For biodiversity, changes in state and function were assessed. Change in state was assessed in terms of: a) change in area in good condition, b) change in land protection and c) change in native biodiversity (considering taxonomic, genetic, phylogenetic, functional, and landscape diversity). Change in function was assessed in terms of: a) change in ecological function (the role that living organisms or groups of organisms play within an ecosystem, contributing to its overall health and stability), b) change in connectivity/fragmentation and c) change in ecological resilience (i.e., the ability to recover structure and function in response to natural or anthropogenic disturbances).

Changes in state and change in function were scored separately, as follows:

- High+ (H+) = increase in at least two component variables and decrease in none
- Medium+ (M+) = increase at least two component variables and decrease in one
- Low+ (L+) = increase at least one component variable and decrease no more than one
- 0 = no change or cancelled out change in the three component variables
- Low- (L-) = decrease in at least one component variables and increase in no more than one
- Medium- (M-) = decrease in at least two component variables and increase in one
- High- (H-) = decrease in at least two component variables and increase in none

Median scores for the two categories were reported.

1. **Impact of the response option on water**

For water, water for humans and water for ecosystems was assessed. For both categories, the extent to which a response option provides or sustains suitable water quality and quantity for humans (and separately for ecosystems) was assessed, for the four conditions: a) provide or maintain suitable water quantity for humans/ecosystems throughout an annual cycle, b) provide or maintain suitable water quantity for humans/ecosystems during critical time periods (seasons), c) provide or maintain suitable water quality for humans/ecosystems throughout an annual cycle and d) provide or maintain suitable water quality for humans/ecosystems during critical time periods (seasons).

Scoring used the following criteria:

- High+ (H+) = increase in at least three of the four component variables and decrease in none
- Medium+ (M+) = increase in at three of the four component variables and decrease in no more than one
- Low+ (L+) = increase in at least two of the four component variables and decrease in no more than one
- 0 = increase in two of the four component variables and decrease in no more than two
- Low- (L-) = increase in one of the four component variables and decrease in no more than one
- Medium- (M-) = increase in one of the four component variables and decrease in two or more
- High- (H-) = No increase in any of the four component variables

Median scores for the four categories were reported.

1. **Impact of the response option on the food system**

The impact of response options of the food system was scored under three categories: a) change in access to healthy food, b) change in environmental performance of food production and c) change in food production (e.g. by increased yield or improved system efficiency), and each was scored as follows:

- High+ (H+) = high positive impact
- Med+ (M+) = medium positive impact
- Low+ (L+) = low positive impact
- 0 = no impact
- Low- (L-) = low negative impact
- Medium- (M-) = medium negative impact
- High- (H-) = high negative impact

Median scores for the three categories were reported.

1. **Impact of the response option on human health & well-being**

The impact of the response options on human health and well-being was assessed according to: a) change in burden of disease (e.g., Years Life Lost) or life lived with disability due to illness or injury (e.g., Disability Adjusted Life Years) or other standard measure, b) change in well-being, including mental, social and cultural well-being, and c) change in access to quality healthcare, including environmentally supportive policies or practice. The first two categories were scored as follows:

- High+ (H+) = high positive impact
- Med+ (M+) = medium positive impact
- Low+ (L+) = low positive impact
- 0 = no impact
- Low- (L-) = low negative impact
- Medium- (M-) = medium negative impact
- High- (H-) = high negative impact

The third category (change in access to quality healthcare, including environmentally supportive policies or practice) was scored as follows:

- High+ (H+) = positive impacts on whole populations/countries
- Medium+ (M+) = positive impacts on remote and vulnerable populations
- Low+ (L+) = positive impacts on vulnerable populations only
- 0 = no impact
- Low- (L-) = negative impacts on vulnerable populations only
- Medium- (M-) = negative impacts on remote and vulnerable populations
- High- (L-) = negative impacts on whole populations/countries

Median scores for the three categories were reported.
